# Association Between Preoperative Rotator Cuff Strength Ratio and Functional Outcomes in Patients with Postoperative Retear After Arthroscopic Rotator Cuff Repair

**DOI:** 10.3390/jcm15135090

**Published:** 2026-06-30

**Authors:** Sang Min Lee, Yong-Gon Seo

**Affiliations:** 1 Division of Sports Medicine, Department of Orthopedic Surgery, Samsung Medical Center, Seoul 06351, Republic of Korea; sangmin1126.lee@samsung.com; 2 Spine Center, Samsung Medical Center, Seoul 06351, Republic of Korea

**Keywords:** rotator cuff repair, postoperative retear, strength ratio, functional recovery

## Abstract

**Background/Objectives**: Structural retear after rotator cuff repair does not always correspond to poor clinical outcomes. Functional factors may help explain this discrepancy and may have rehabilitation relevance in patients with postoperative retear. This study aimed to evaluate the association of preoperative external-to-internal rotation strength ratio for postoperative pain and functional outcomes in patients with retear after arthroscopic rotator cuff repair. **Methods**: This retrospective cohort study included 72 patients who underwent arthroscopic rotator cuff repair at a tertiary referral center between January 2015 and December 2019 and whose magnetic resonance imaging performed 5–6 months after surgery revealed postoperative retear. Patients were classified according to the preoperative external-to-internal rotation strength ratio into a normal group (NG; ratio, 60–80%; *n* = 34) and an abnormal group (AG; *n* = 38). Postoperative outcomes were assessed at 1 year and 2 years using functional and pain visual analog scale (FVAS and PVAS), the American Shoulder and Elbow Surgeons (ASES) score, and Constant score. Two-way repeated measures analysis of variance (ANOVA) was used to assess group and time effects and group × time interaction. **Results**: No significant between-group differences were observed at baseline for PVAS and FVAS scores, ASES score, or Constant score. During follow-up, NG showed significantly lower PVAS values at 1 year (*p* = 0.007) and 2 years (*p* = 0.009), significantly higher FVAS scores at 1 year (*p* = 0.002) and 2 years (*p* = 0.007), significantly higher Constant scores at 1 year (*p* = 0.019) and 2 years (*p* = 0.020), and significantly higher ASES scores at 2 years than the AG (*p* = 0.041). Repeated-measures ANOVA demonstrated significant time effects for all outcome measures (all *p* < 0.001) and significant group effects for PVAS (*p* = 0.007) and FVAS scores (*p* = 0.002) and ASES (*p* = 0.016) and Constant scores (*p* = 0.012). No significant group × time interaction was observed for any outcome measure. **Conclusions**: A normal preoperative external-to-internal rotation strength ratio was associated with better pain and functional outcomes in patients with structurally confirmed postoperative retear after arthroscopic rotator cuff repair. These findings suggest that preoperative rotator cuff strength balance may provide clinically useful information for postoperative functional stratification.

## 1. Introduction

Rotator cuff tears are a common cause of shoulder pain and dysfunction and may substantially impair daily activities and quality of life [1]. When nonoperative treatment fails to relieve symptoms or restore function, rotator cuff repair is widely performed as an effective surgical treatment [2]. Despite advances in surgical techniques and postoperative rehabilitation protocols, postoperative structural failure remains a relatively common problem after rotator cuff repair [3,4].

Retear after rotator cuff repair has generally been considered a negative prognostic factor because it reflects failed tendon healing and may adversely affect postoperative pain relief and functional recovery [3]. However, previous studies have shown that radiologic evidence of retear does not invariably lead to poor clinical outcomes. Some patients with postoperative retears continue to demonstrate satisfactory pain relief and functional improvement despite structural failure [5]. These findings suggest that anatomic failure does not always correspond to clinical failure and that the clinical significance of retear may vary according to individual patient characteristics [6].

To explain this discrepancy, various prognostic factors, including patient age, fatty degeneration, and muscle quality, have been investigated [7]. In addition, the preoperative functional status of the shoulder may influence postoperative clinical outcomes. In particular, the strength of internal and external rotator muscles is closely related to glenohumeral stability and dynamic shoulder function and may reflect the functional integrity and balance of the rotator cuff [8].

The external-to-internal rotation strength ratio represents the relative balance between the shoulder external and internal rotator muscles, rather than the absolute strength of either muscle group alone. From a biomechanical perspective, balanced activation of the rotator cuff muscles is important for dynamic glenohumeral stability because these muscles contribute to humeral head centralization during shoulder motion [9]. Therefore, the ER/IR strength ratio may serve as a functional indicator of the shoulder force couple and rotator cuff strength balance. Previous studies using handheld dynamometry have shown that this ratio can vary depending on the testing position, measurement method, and participant characteristics, suggesting that it should be interpreted within a specific clinical and methodological context [10]. Clinically, a more balanced preoperative rotator cuff strength ratio has been associated with better postoperative shoulder function after rotator cuff repair, supporting its potential relevance as a preoperative functional marker [11].

A normal preoperative external-to-internal rotation strength ratio may reflect relatively preserved rotator cuff function and muscular balance, which may in turn contribute to more favorable clinical outcomes despite postoperative retear [12]. Unlike static structural variables, the preoperative external-to-internal rotation strength ratio may provide clinically meaningful information regarding functional muscle balance of the shoulder and may therefore be useful in interpreting postoperative recovery, functional prognosis, and rehabilitation needs in patients with postoperative retear [11].

Nevertheless, the existing literature has focused primarily on the association between structural integrity and postoperative clinical outcomes after rotator cuff repair, with limited attention given to prognostic factors within the subgroup of patients who develop retears [13]. In particular, the predictive significance of the preoperative rotator cuff strength ratio in patients with postoperative retear has not been sufficiently investigated. Therefore, the purpose of this study was to evaluate the association between the preoperative external-to-internal rotation strength ratio and postoperative pain and functional outcomes in patients with retear after arthroscopic rotator cuff repair.

## 2. Materials and Methods

### 2.1. Study Design

This retrospective cohort study was conducted to evaluate the predictive value of the preoperative external-to-internal rotation strength ratio in patients with postoperative retear after arthroscopic rotator cuff repair. Postoperative clinical outcomes were evaluated at 1 year and 2 years after surgery using the pain visual analog scale (PVAS), functional visual analog scale (FVAS), the American Shoulder and Elbow Surgeons (ASES) score, and Constant score.

### 2.2. Participants

**Figure 1:**
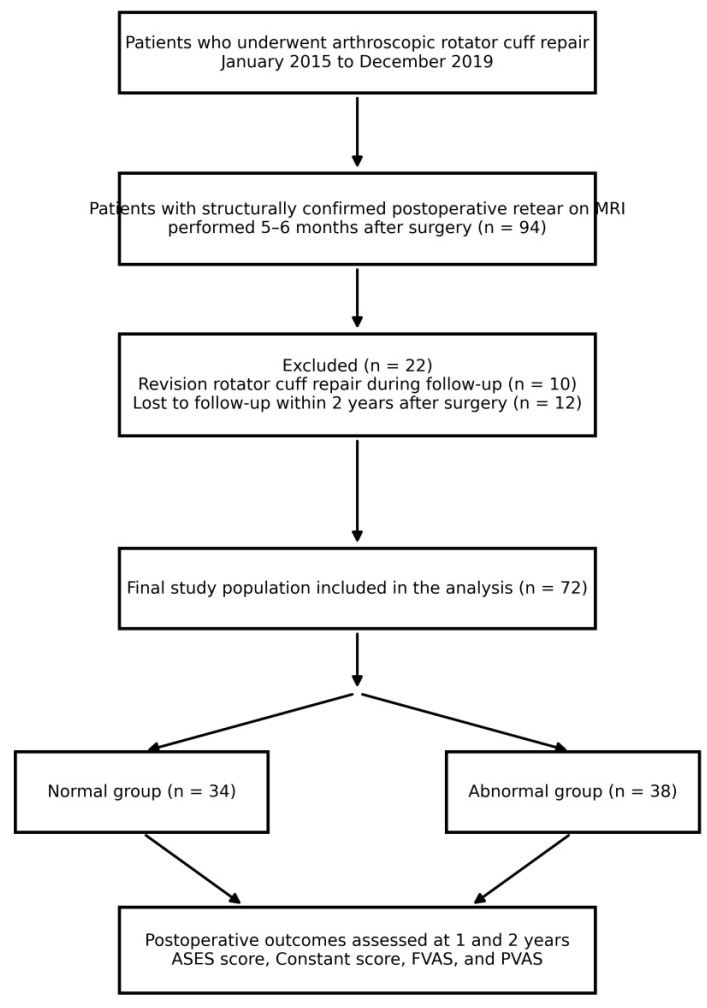
Flowchart of patient selection and group allocation.

We retrospectively reviewed patients who underwent arthroscopic rotator cuff repair between January 2015 and December 2019 at our institution. Among them, patients who showed radiologic evidence of retear on magnetic resonance imaging (MRI) performed 5 to 6 months after surgery were identified. Initially, 94 patients with postoperative retear were eligible for inclusion. The postoperative structural integrity of the repaired rotator cuff was evaluated on MRI using the Sugaya classification system. Tendon integrity was classified into five types: type I, repaired cuff with sufficient thickness and homogeneous low intensity on each image; type II, sufficient thickness with partial high-intensity area; type III, insufficient thickness without discontinuity; type IV, minor discontinuity in more than one slice, suggestive of a small tear; type V, major discontinuity in each image, indicating a medium or large tear [14]. In the present study, Sugaya types I, II, and III were regarded as healed tendons, whereas Sugaya types IV and V were defined as retears. Accordingly, only patients with Sugaya type IV or V on postoperative MRI performed 5 to 6 months after surgery were included in the study. All arthroscopic rotator cuff repairs were performed by a single senior orthopedic surgeon using a standardized technique. The procedure was performed with the patient in the lateral decubitus position under general anesthesia. After diagnostic arthroscopy, the torn rotator cuff was repaired using a transosseous-equivalent suture-bridge technique. Concomitant procedures, including subacromial decompression and biceps tenotomy or tenodesis, were performed when indicated. Patients were classified into two groups according to the preoperative external-to-internal rotation strength ratio. Patients with a ratio between 60% and 80% were assigned to the normal group (NG), whereas those with a ratio outside this range were assigned to the abnormal group (AG) [15,16]. Patients who underwent revision rotator cuff repair during follow-up (*n* = 10) or were lost to follow-up within 2 years after surgery (*n* = 12) were excluded. After applying these exclusion criteria, 72 patients were included in the final analysis (NG, *n* = 34; AG, *n* = 38) (Figure 1).

### 2.3. Preoperative Strength Assessment

**Figure 2:**
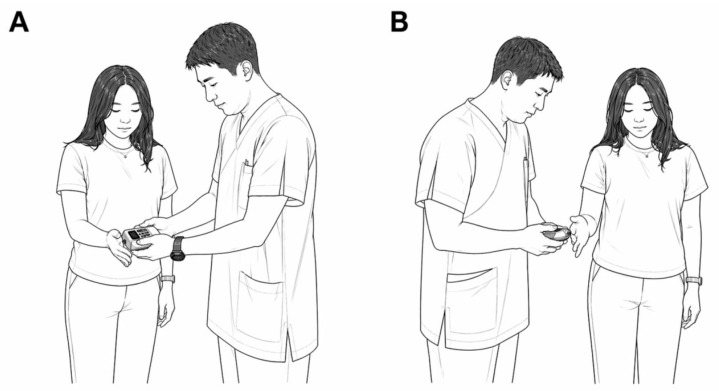
Measurement of preoperative shoulder muscle strength using a handheld dynamometer: (**A**) internal rotation and (**B**) external rotation.

Preoperative shoulder muscle strength was assessed by measuring maximal isometric strength using a handheld dynamometer (FGN-20B; Nidec Shimpo, Kyoto, Japan). Measurements were performed with the patient maintaining the arm adducted to the side of the trunk and the elbow flexed at 90°. Internal rotation strength was measured after instructing the patient to perform shoulder internal rotation, with the dynamometer placed on the volar aspect of the forearm. External rotation strength was measured after instructing the patient to perform shoulder external rotation, with the dynamometer placed on the dorsal aspect of the forearm [10] (Figure 2).

Each measurement was repeated under identical conditions, and the mean value was used for analysis. To evaluate the muscular balance of the affected shoulder, the external-to-internal rotation strength ratio was calculated by dividing external rotation strength by internal rotation strength. Isometric strength of internal and external rotation was assessed only in the affected shoulder, and the absolute strength values were not normalized to body weight or contralateral shoulder strength. The external-to-internal rotation strength ratio was calculated as external rotation strength divided by internal rotation strength on the affected shoulder. Based on this ratio, patients were classified into either the normal group (NG) or the abnormal group (AG) according to a predefined normal range of 60–80%. A normal external-to-internal rotation strength ratio has been reported to approximate a 2:3 relationship, with the external rotators producing about two-thirds of internal rotator strength in healthy individuals [15,16], and a balanced ratio between the external and internal rotators is considered essential for maintaining dynamic centering of the humeral head [16]. We therefore adopted a normal range of 60–80% centered on these reported values, allowing for individual variability and measurement variability while capturing the concept of a balanced force couple. This range was also consistent with our previous study of patients undergoing rotator cuff repair, in which a comparable preoperative strength-ratio range was used to define normal rotator cuff balance [11]. Accordingly, patients whose ratio fell within 60–80% were classified as NG, reflecting a preserved force couple, whereas those whose ratio fell outside this range either below 60% or above 80% were classified as AG, reflecting a disrupted force couple. Because the strength ratio expresses the relative balance between the two muscle groups within the same shoulder, it is less affected by age-related reductions in absolute strength than are absolute strength values [17]. To minimize measurement bias and interobserver variability, all strength and postoperative functional assessments were conducted by the same experienced physical trainer, who was blinded to the patients’ group allocation and postoperative MRI findings.

### 2.4. Postoperative Rehabilitation

All patients followed an identical standardized postoperative rehabilitation protocol regardless of group allocation. The operated arm was immobilized in an abduction brace for 4–6 weeks. Passive range-of-motion exercises were initiated at 2–4 weeks, active-assisted motion at 4–6 weeks, and active motion at 6–8 weeks postoperatively. Strengthening exercises were initiated at postoperative weeks 10–12. The same protocol was applied to both groups.

### 2.5. Outcome Assessment

Postoperative clinical outcomes were assessed at 1 year and 2 years after surgery using the PVAS, FVAS, ASES score, and Constant score.

The PVAS was used to assess subjective pain intensity. Pain was scored on a scale from 0 to 10, where 0 represented no pain and 10 represented the worst imaginable pain. Lower scores indicate less pain [18]. The FVAS was used to evaluate the patient’s subjective perception of shoulder functional status. Function was rated on a scale from 0 to 10, where 0 indicated severe functional impairment and 10 indicated normal function without limitation. Higher scores indicate better perceived shoulder function [19]. The ASES score was used to assess shoulder pain and functional disability. It consists of a pain component (50 points) and a functional component (50 points). The functional component includes 10 items related to activities of daily living, and the total score ranges from 0 to 100, with higher scores indicating better function [20]. The Constant score was used to assess overall shoulder function. It is composed of pain (15 points), activities of daily living (20 points), range of motion (40 points), and strength (25 points), with a maximum total score of 100 points and higher scores indicating better shoulder function [21].

### 2.6. Statistical Analyses

Statistical analyses were performed using SPSS version 21.0 (IBM Corp., Armonk, NY, USA). Continuous variables are expressed as mean ± standard deviation, and categorical variables are presented as frequencies with percentages. Baseline demographic and clinical characteristics were compared between NG and AG using the independent *t*-test for continuous variables and the chi-square test or Fisher’s exact test for categorical variables, as appropriate.

Longitudinal changes in postoperative clinical outcomes, including the PVAS scores, FVAS scores, ASES scores, and Constant scores, were analyzed using two-way repeated measures analysis of variance (ANOVA), with group as the between-subject factor and time as the within-subject factor. Group effect, time effect, and group × time interaction were assessed. When significant main effects were identified, between-group comparisons at each time point were performed using independent *t*-tests. Bonferroni correction was applied for multiple comparisons, where appropriate. The assumption of sphericity was assessed using Mauchly’s test, and Greenhouse–Geisser correction was applied when the sphericity assumption was violated. A *p*-value of <0.05 was considered statistically significant.

## 3. Results

### 3.1. Demographic and Clinical Characteristics

Baseline demographic and clinical characteristics are presented in Table 1. No significant between-group differences were observed in age (*p* = 0.759), height (*p* = 0.900), weight (*p* = 0.663), sex (*p* = 0.554), dominant arm (*p* = 0.580), injury side (*p* = 0.941), tear size (*p* = 0.562), or diabetes mellitus (*p* = 0.867), indicating that NG and AG were comparable with respect to baseline demographic and clinical characteristics.

### 3.2. Pain Visual Analog Scale

The PVAS scores at each time point are presented in Table 2. There was no significant between-group difference at baseline (*p* = 0.489). However, NG showed significantly lower PVAS scores than AG at 12 (*p* = 0.007) and 24 months (*p* = 0.009).

**Figure 3:**
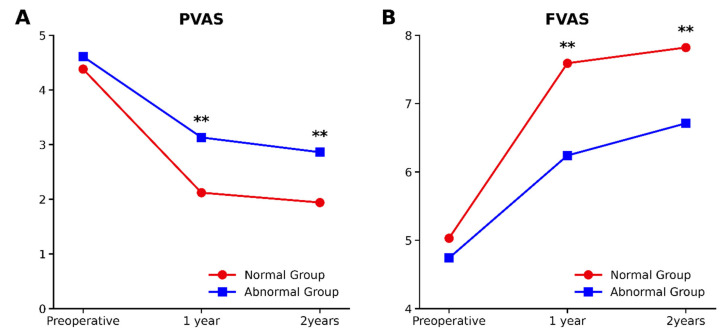
Longitudinal changes in clinical outcomes during follow-up: (**A**) pain visual analog scale (PVAS) and (**B**) functional visual analog scale (FVAS). ** *p* < 0.01, between groups at each time point.

Repeated-measures ANOVA demonstrated a significant main effect of time (*F* = 69.516, *p* < 0.001) and a significant main effect of group (*F* = 7.792, *p* = 0.007), whereas the group × time interaction was not significant (*F* = 2.438, *p* = 0.091) (Table 2 and Figure 3).

### 3.3. Functional Visual Analog Scale

The FVAS scores at each time point are shown in Table 2. No significant between-group difference was observed at baseline (*p* = 0.502). In contrast, NG demonstrated significantly higher FVAS scores than AG at 12 (*p* = 0.002) and 24 months (*p* = 0.007). Repeated-measures ANOVA revealed a significant main effect of time (Greenhouse–Geisser corrected *F* = 45.760, *p* < 0.001) and a significant main effect of group (*F* = 10.578, *p* = 0.002), whereas the group × time interaction was not significant (*F* = 2.133, *p* = 0.137) (Table 2 and Figure 3).

### 3.4. American Shoulder and Elbow Surgeons Score

**Figure 4:**
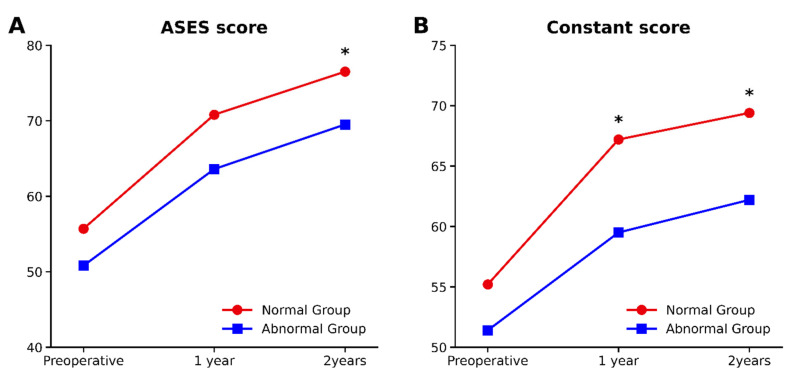
Longitudinal changes in clinical outcomes during follow-up: (**A**) American Shoulder and Elbow Surgeons (ASES) score. (**B**) Constant score. * *p* < 0.05, between groups at each time point.

The ASES scores at each time point are summarized in Table 2. There was no significant between-group difference at baseline (*p* = 0.152). At 12 months, the difference between the two groups did not reach statistical significance (*p* = 0.062). However, NG had significantly higher ASES scores than AG at 24 months (*p* = 0.041). Repeated-measures ANOVA showed a significant main effect of time (Greenhouse–Geisser corrected *F* = 47.930, *p* < 0.001) and a significant main effect of group (*F* = 6.105, *p* = 0.016), whereas the group × time interaction was not significant (*F* = 0.198, *p* = 0.794) (Table 2 and Figure 4).

### 3.5. Constant Score

The Constant scores at each time point are presented in Table 2. No significant between-group difference was observed at baseline (*p* = 0.271). NG demonstrated significantly higher Constant scores than AG at 12 (*p* = 0.019) and 24 months (*p* = 0.020). Repeated-measures ANOVA demonstrated a significant main effect of time (Greenhouse–Geisser corrected *F* = 25.516, *p* < 0.001) and a significant main effect of group (*F* = 6.705, *p* = 0.012), whereas the group × time interaction was not significant (*F* = 0.657, *p* = 0.479) (Table 2 and Figure 4).

## 4. Discussion

The principal finding of the present study was that, among patients with structurally confirmed postoperative retear after arthroscopic rotator cuff repair, those with a normal preoperative external-to-internal rotation strength ratio had better overall pain and functional outcomes during follow-up. Although both groups showed significant improvement over time in PVAS and FVAS scores, ASES scores, and Constant scores, NG maintained superior overall clinical scores during follow-up. However, the absence of a significant group × time interaction for all outcome measures indicates that the longitudinal pattern of recovery was similar between the groups. In other words, patients with a normal preoperative rotator cuff strength ratio did maintain a more favorable overall level of pain relief and functional outcome throughout follow-up.

These findings support previous reports that structural failure after rotator cuff repair does not necessarily correspond to poor clinical outcome [22,23]. The present study further suggests that preoperative muscular balance, reflected by the external-to-internal rotation strength ratio may partly explain why some patients maintain satisfactory pain relief and function despite postoperative retear.

This association may be explained, at least in part, by the biomechanical role of balanced internal and external rotator strengths in dynamic glenohumeral stability [24,25]. The internal and external rotator muscles do not function solely as generators of rotational movement; rather, they contribute to maintaining the humeral head centered within the glenoid throughout motion [12]. From this perspective, both an excessively low and an excessively high strength ratio represent a loss of the balanced force couple rather than two opposite favorable conditions; a ratio above the normal range reflects relative internal rotator deficiency, just as a ratio below the normal range reflects relative external rotator weakness. This conceptual framework supports grouping both deviations together as an abnormal, imbalanced state in the present analysis. Accordingly, a normal preoperative external-to-internal rotation strength ratio may indicate better functional coordination of the shoulder musculature and a more preserved dynamic stabilizing mechanism [11]. Such muscular balance may facilitate more efficient humeral head centralization, improve dynamic control of subtle joint motion, and support better movement quality during postoperative recovery. Even when structural retear occurs, patients with better preoperative muscular balance may retain greater functional reserve and compensatory capacity, which may partly explain their more favorable pain and functional outcomes during follow-up [11,26]. Thus, patients in whom the balance between the internal and external rotators is well preserved before surgery may be more likely to maintain favorable clinical outcomes despite postoperative retear.

In addition, the present findings may also be interpreted in relation to scapular function. The scapula provides a stable base for glenohumeral motion, and normal scapulothoracic rhythm and appropriate scapular movement are essential for efficient humeral motion and maintenance of shoulder function [27]. Conversely, scapular dysfunction may alter shoulder kinematics and contribute to pain, weakness, and functional limitation [28,29]. From this perspective, a normal preoperative external-to-internal rotation strength ratio may reflect not only balanced rotator cuff strength but also relatively preserved neuromuscular control of the shoulder complex, including scapular function [30]. Therefore, the better postoperative outcomes observed in NG may partly reflect the beneficial influence of preexisting muscular balance and coordinated shoulder motion during postoperative rehabilitation and functional recovery.

Another notable finding was that no significant between-group difference was observed preoperatively in PVAS scores and FVAS scores, ASES scores, and Constant scores, whereas significant between-group differences emerged during follow-up in most outcomes. Specifically, NG demonstrated lower PVAS scores, higher FVAS scores, and higher Constant scores at both 1 year and 2 years, whereas the difference in ASES scores reached statistical significance only at 2 years. This pattern suggests that the clinical relevance of preoperative muscular balance may become more evident during postoperative recovery rather than being fully reflected in preoperative symptoms or self-reported functional status. The delayed between-group difference in ASES scores should be interpreted cautiously, but it may indicate that some functional differences become more apparent over time [22,31].

The clinical relevance of the observed between-group differences should be interpreted in light of previously reported minimal clinically important difference (MCID) values. In the present study, the between-group differences at 1 and 2 years were 1.01 and 0.92 points for PVAS, 1.35 and 1.11 points for FVAS, 7.13 and 7.11 points for the ASES score, and 7.71 and 7.17 points for the Constant score, respectively. Previous studies after rotator cuff repair have reported MCID values of approximately 1.5–2.4 points for pain VAS [9] and 11.1–27.1 points for the ASES score [18]. Therefore, although statistically significant differences were observed in PVAS and ASES at follow-up, these differences did not clearly exceed previously reported MCID thresholds. For the Constant score, the observed differences exceeded the lower MCID value of 4.6 points reported [32] after arthroscopic rotator cuff repair but did not exceed the more conservative threshold of 10.4 [33] points reported in patients with rotator cuff tears. Because a rotator cuff repair-specific MCID for FVAS is not firmly established, the clinical meaning of the FVAS difference should be interpreted with caution. Overall, these findings suggest that preoperative ER/IR strength balance may be associated with statistically detectable differences in postoperative outcomes; however, the clinical magnitude of the between-group differences may be modest. Nevertheless, both groups showed substantial improvement from baseline to the 2-year follow-up in pain and functional outcomes, suggesting that arthroscopic rotator cuff repair provided clinically meaningful improvement regardless of preoperative ER/IR strength ratio status. The normal ratio group tended to show more favorable postoperative scores, but the between-group differences should be regarded as modest when interpreted against established MCID thresholds.

The lack of a significant group × time interaction also deserves careful interpretation. At first glance, this finding may appear to suggest that the preoperative strength ratio does not influence postoperative recovery. However, the significant main effect of group across all outcome measures indicates that NG consistently maintained better clinical status throughout follow-up. Therefore, the present findings should not be interpreted as evidence that preoperative strength ratio is unrelated to outcome, but rather that it is associated with the overall level of postoperative pain and function rather than with a distinctly different recovery trajectory. Clinically, this means that patients with a normal preoperative strength ratio may be expected to maintain a better overall postoperative condition despite retear, even if the shape of their recovery curve is similar to that of patients with an abnormal ratio. To better contextualize the present findings, previous studies on rotator cuff strength balance, shoulder stabilization mechanisms, sports-related shoulder adaptation, and postoperative retear outcomes are summarized in Table 3 [11,15,26,30,34,35,36,37]. Biomechanical studies have shown that the rotator cuff muscles contribute to dynamic glenohumeral stability by maintaining humeral head centralization during shoulder motion [30]. In this context, the external-to-internal rotation strength ratio may reflect not only isolated muscle strength but also the functional balance of the shoulder force couple [10,34]. Previous clinical studies have suggested that preoperative rotator cuff strength balance is associated with postoperative shoulder function after rotator cuff repair [11]. However, the interpretation of this ratio should consider the measurement position, population characteristics, and physical activity demands [10].

In particular, sports activity may influence intrinsic shoulder stabilization mechanisms. Overhead or shoulder-demanding sports can lead to sport-specific adaptations in internal and external rotator strength, scapular control, proprioception, and neuromuscular coordination [26,37]. Therefore, the type and level of sport or physical activity may affect the external-to-internal rotation strength ratio and postoperative functional recovery [10,26,37]. Although the present study focused on patients with structurally confirmed postoperative retear, detailed information regarding sports participation and activity level was not available. This should be considered when interpreting the association between preoperative rotator cuff strength balance and postoperative outcomes [26,30].

This study has several limitations. First, the retrospective design introduces the potential for selection bias and limits causal inference. Second, this was a single-center study, and all procedures were performed by a single senior surgeon, which may limit the generalizability of the findings. Third, only patients with MRI-confirmed retear at 5–6 months after surgery were included; thus, the results may not be applicable to the overall population of patients undergoing rotator cuff repair. Fourth, postoperative outcomes were evaluated mainly using patient-reported and clinician-based functional scores, without additional performance-based measures or return-to-activity outcomes. Fifth, because multivariable analysis was not performed, the independent prognostic value of the preoperative strength ratio could not be confirmed, as the influence of potential confounders such as age, sex, tear size, and fatty infiltration could not be fully assessed; detailed information on the type, intensity, and level of sports or physical activity was likewise unavailable.

Several limitations also relate specifically to the strength ratio. The 60–80% normal range was based partly on normative data from younger or general populations, as no reference specific to elderly patients with postoperative retear exists. The abnormal group also combined low (<60%) and high (>80%) ratios; the small high-ratio subgroup precluded reliable stratified analysis, warranting separate evaluation in larger studies. In addition, forward elevation strength was not included in the assessment of shoulder muscle function; because forward elevation reflects an important component of functional shoulder performance, the external-to-internal rotation strength ratio may not fully capture overall shoulder strength or multidirectional shoulder function.

Accordingly, the present results should be interpreted as demonstrating an association between preoperative strength ratio and postoperative outcomes rather than confirming an independent prognostic effect. The present study has several clinical implications for postoperative rehabilitation and patient counseling. First, preoperative assessment of internal and external rotation strength using a handheld dynamometer is simple and feasible in routine clinical practice. A normal preoperative strength ratio may provide useful functional information when counseling patients regarding expected postoperative outcomes after rotator cuff repair. Second, in patients with postoperative retear, MRI findings alone may be insufficient in estimating functional prognosis, and preoperative muscular balance should also be taken into consideration. Third, patients with an abnormal preoperative strength ratio may benefit from closer postoperative monitoring and more individualized rehabilitation strategies targeting muscular balance, dynamic shoulder control, and functional compensation.

## 5. Conclusions

Among patients with postoperative retear after arthroscopic rotator cuff repair, those with a normal preoperative external-to-internal rotation strength ratio demonstrated better overall pain and functional outcomes during follow-up. Although the pattern of recovery over time was similar between groups, preoperative rotator cuff strength balance may provide clinically useful information for estimating postoperative clinical status in this population. However, the present findings should be interpreted as demonstrating an association between preoperative rotator cuff strength balance and postoperative outcomes, rather than confirming an independent predictive effect.

## Figures and Tables

**Table 1:** Demographic characteristics of the patients (*n* = 72).

Variables	Normal Group (*n* = 34)	Abnormal Group (*n* = 38)	*p*-Value
Age (years)	61.32 ± 7.15	60.82 ± 6.80	0.759
Height (cm)	158.71 ± 7.26	158.62 ± 6.97	0.900
Weight (kg)	61.60 ± 9.70	63.90 ± 9.87	0.663
Sex			0.554
Male	13 (38.2)	15 (39.5)	
Female	21 (61.8)	23 (60.5)	
Dominant arm			0.580
Right	30 (88.2)	35 (92.1)	
Left	4 (11.8)	3 (7.9)	
Injury side			0.941
Right	29 (85.3)	32 (84.2)	
Left	5 (14.7)	6 (15.8)	
Tear size			0.562
Small	4 (11.8)	3 (7.9)	
Medium	12 (35.3)	18 (47.4)	
Large	18 (52.9)	17 (44.7)	
Diabetes mellitus	4 (11.8)	4 (10.5)	0.867
Muscle strength			
External rotation (lbs)	10.27 ± 4.22	7.73 ± 3.90	0.010
Internal rotation (lbs)	14.42 ± 5.80	14.55 ± 5.57	0.919
ER/IR ratio	71.51 ± 7.44	58.24 ± 31.56	0.019

Values are presented as mean ± standard deviation or *n* (%).

**Table 2:** Result of repeated-measures analysis of variance for pain and functional outcomes.

Variables	Follow-Up	Normal Group (*n* = 34)	Abnormal Group (*n* = 38)	*p*-Value	Variables	*F*	*p*-Value
PVAS	Preoperative	4.38 ± 1.30	4.61 ± 1.41	0.489	Time	69.516	<0.001 ***
	1 year	2.12 ± 1.47	3.13 ± 1.61	0.007 **	Group × Time	2.438	0.091
	2 years	1.94 ± 1.20	2.86 ± 1.61	0.009 **	Group	7.792	0.007 **
FVAS	Preoperative	5.03 ± 1.78	4.74 ± 1.88	0.502	Time	45.760	<0.001 ***
	1 year	7.59 ± 1.35	6.24 ± 2.12	0.002 **	Group × Time	2.133	0.137
	2 years	7.82 ± 1.17	6.71 ± 2.05	0.007 **	Group	10.578	0.002 **
ASES score	Preoperative	55.71 ± 14.36	50.84 ± 14.12	0.152	Time	47.930	<0.001 ***
	1 year	70.74 ± 14.32	63.61 ± 17.19	0.062	Group × Time	0.198	0.794
	2 years	76.56 ± 11.49	69.45 ± 16.71	0.041 *	Group	6.105	0.016 *
Constant score	Preoperative	55.18 ± 15.23	51.39 ± 13.68	0.271	Time	25.516	<0.001 ***
	1 year	67.21 ± 12.96	59.50 ± 14.23	0.019 *	Group × Time	0.657	0.479
	2 years	69.38 ± 11.78	62.21 ± 13.63	0.020 *	Group	6.705	0.012 *

Values are presented as mean ± standard deviation. PVAS, pain visual analog scale; FVAS, functional visual analog scale; ASES, American Shoulder and Elbow Surgeons. * *p* < 0.05, ** *p* < 0.01, *** *p* < 0.001.

**Table 3:** Summary of relevant literature on rotator cuff strength balance, shoulder stability, sports activity, and postoperative retear outcomes.

Study	Study Design/Population	Main Methodology	Key Findings	Clinical Implication
Riemann et al., 2010 [10]	Normative study using handheld dynamometry	Measured internal and external rotator strength in different testing positions	ER/IR ratios varied according to testing position and participant characteristics	Supports the use of handheld dynamometry, while emphasizing that strength ratios require context-specific interpretation
Lee et al., 2020 [11]	Clinical cohort study after rotator cuff repair	Evaluated the association between preoperative rotator muscle strength ratio and postoperative shoulder function	A normal preoperative rotator muscle strength ratio was associated with better postoperative shoulder function	Directly supports the clinical relevance of preoperative rotator cuff strength balance
Ackland et al., 2009 [34]	Anatomical and biomechanical study	Analyzed lines of action and stabilizing potential of major shoulder muscles during abduction and flexion	Shoulder muscles showed different stabilizing roles depending on the direction and plane of motion	Supports the concept that shoulder stability depends on coordinated muscle function rather than isolated strength alone
Burn et al., 2016 [35]	Systematic review in overhead and non-overhead athletes	Compared the prevalence of scapular dyskinesis between athlete groups	Scapular dyskinesis was more prevalent in overhead athletes than in non-overhead athletes	Indicates that sports activity may affect scapular control and shoulder stabilization mechanisms
Hickey et al., 2018 [36]	Systematic review and meta-analysis in asymptomatic athletes	Evaluated whether scapular dyskinesis increases the risk of future shoulder pain	Scapular dyskinesis was associated with an increased risk of future shoulder pain	Supports the clinical relevance of scapular control as an intrinsic factor in shoulder function
Holtedahl et al., 2023 [37]	Systematic review and meta-analysis	Analyzed the clinical impact of retears after repair of posterosuperior rotator cuff tears	Retear was associated with differences in pain and function, although clinical interpretation remains complex	Reinforces the need to evaluate additional functional factors in patients with retear
Kim et al., 2023 [26]	Retrospective cohort study	Compared clinical outcomes, strength recovery, return to work, and return to sports according to retear status	Retear influenced some functional and activity-related outcomes	Highlights the importance of activity-related outcomes after rotator cuff repair
Lee et al., 2025 [30]	Clinical study comparing healed and retear groups after arthroscopic rotator cuff repair	Assessed muscle strength, neuromuscular control, and patient-reported outcomes	Neuromuscular control and functional outcomes may provide additional information beyond structural integrity	Supports the need to consider neuromuscular and functional factors in patients with postoperative retear

ER, external rotation; IR, internal rotation.

## Data Availability

The data that support the findings of this study are not openly available due to reasons of sensitivity and are available from the corresponding author upon reasonable request.
